# Protective Effect of the Hexanic Extract of *Eryngium carlinae* Inflorescences In Vitro, in Yeast, and in Streptozotocin-Induced Diabetic Male Rats

**DOI:** 10.3390/antiox8030073

**Published:** 2019-03-26

**Authors:** Donovan J. Peña-Montes, Maribel Huerta-Cervantes, Mónica Ríos-Silva, Xóchitl Trujillo, Miguel Huerta, Ruth Noriega-Cisneros, Rafael Salgado-Garciglia, Alfredo Saavedra-Molina

**Affiliations:** 1 Instituto de Investigaciones Químico Biológicas, Universidad Michoacana de San Nicolás de Hidalgo, 58030 Morelia, Mich., Mexico; yodonnie@gmail.com (D.J.P.-M.); marzy112@yahoo.com.mx (M.H.-C.); rsalgadogarciglia@gmail.com (R.S.-G.); 2 Centro Universitario de Investigaciones Biomédicas, Universidad de Colima, 28040 Colima, Col., Mexico; mrios@ucol.mx (M.R.-S.); rosio@ucol.mx (X.T.); huertam@ucol.mx (M.H.); 3 Facultad de Enfermería, Universidad Michoacana de San Nicolás de Hidalgo, 58260 Morelia, Mich., Mexico; ruthnc26@yahoo.com

**Keywords:** antioxidant activity, diabetes mellitus, *Eryngium carlinae*, oxidative stress, *Saccharomyces cerevisiae*

## Abstract

In the present study, we investigated the composition and antioxidant activity of the hexanic extract of *Eryngium carlinae* inflorescences by employing in vitro assays to measure antioxidant capacity and 2,2-diphenyl-1-picrylhydrazyl scavenging activity. We also applied the hexanic extract to *Saccharomyces cerevisiae*, under hydrogen peroxide-induced stress. Finally, we tested the extract in male Wistar rats with and without streptozotocin-induced diabetes. The compounds in the hexanic extract were analyzed by gas-chromatography-mass spectrometry, which revealed mainly terpenes and sesquiterpenes, including (Z)β-farnesene (38.79%), β-pinene (17.53%), calamene (13.3%), and α-farnesene (10.38%). In vitro and in *S. cerevisiae*, the extract possessed antioxidant activity at different concentrations, compared to ascorbic acid (positive control). In normoglycemic and hyperglycemic rats, oral administration of 30 mg/kg of the extract reduced blood glucose levels; lipid peroxidation in liver, kidney and brain; protein carbonylation; and reactive oxygen species (ROS) production. It also increased catalase activity in the brain, kidneys and liver. These findings show that this hexanic extract of *E. carlinae* inflorescences possessed antioxidant properties.

## 1. Introduction

Diabetes mellitus (DM) comprises a heterogeneous set of multifactorial pathogenetic syndromes. The common nexus is a metabolic disorder, which mainly includes chronic hyperglycemia, but also alterations in lipid and protein metabolism. Chronic hyperglycemia in the diabetic milieu contributes to the development of complications in multiple organs and tissues. Current evidence has revealed the many complications associated with DM, including heart attacks, strokes, renal failure, leg amputation, loss of vision, and neurological damage [1]. Diabetes treatment is costly for many people in developing countries, and such medical expenses represent an additional burden. This population often relies on the alternative therapy of medicinal plants for the treatment of various disorders. There are several reports about the beneficial effects of a wide range of plants to treat diabetes. Antioxidant supplementation derived from food and medicinal plants has been increasingly investigated for its various nutritional functions and health benefits in chronic diseases, such as DM [2]. An external supply of antioxidants can counteract the effect of reactive oxygen species (ROS) in the body and, in turn, prevent the emergence of many underlying diseases and complications [3]. Therefore, considerable attention has been focused on the analysis, isolation, characterization, and use of natural antioxidants as prevention agents.

Plants are one of the most promising sources of new antioxidant agents [4]. Several types of plants have been used, both as dietary supplements and as traditional medicines, for treating various health problems [5]. *Eryngium carlinae* (EC) is a traditional herbal medicine in Mexico, commonly known as “Frog herb”. *Eryngium carlinae* has been attributed with diuretic and healing properties. Previous research has shown that species of this genus contain a wide variety of secondary metabolites with bioactive potential, including flavonoids, saponines, triterpenes, coumarins, and derivatives of rosmarinic acid, polyacetylenes, and essential oils [6]. Currently, oral EC is taken daily in water to regulate blood pressure and lower blood lipids. It has also been prepared as a decoction or ethanolic extract. In previous studies, a hexanic and ethanol extracts of EC were shown to possess hypoglycemic and hypolipidemic activity in diabetic rats [7,8,9,10]. The present study aimed to investigate the chemical composition and major compounds contained in the hexanic extract of *E. carlinae* inflorescences with gas chromatography/mass spectrometry (GC/MS) and to determine the antioxidant capacity of the hexanic extract in vitro and in vivo, in both *Saccharomyces cerevisiae* and streptozotocin-induced diabetic rats.

## 2. Materials and Methods 

### 2.1. Plant Material

Dried inflorescences of *E. carlinae* (200 g) were triturated and macerated with *n*-hexane (triturated plant-to-solvent ratio: 1:10 in *n*-hexane) and stored at 4 °C for seven days. Then, the extract was filtered and concentrated in a rotary evaporator under vacuum at ≤30 °C. This hexanic extract was stored in the dark at 4 °C until use.

### 2.2. Hexanic Extract Characterization with GC/MS

The hexanic extract was chemically characterized on a 7890 gas chromatograph (Agilent Technologies, Palo Alto, CA, USA) configured with an Agilent 7693A automatic liquid sampler (Agilent Technologies, Palo Alto, CA, USA) and coupled to an Agilent 5975C Series gas chromatograph/mass selective detector (Agilent Technologies, Palo Alto, CA, USA). The following parameters were applied: split injection (50:1) at 220 °C, using helium as the carrier gas, and a column flow of 1 mL/min, in a non-polar HP 5MS (5% phenyl 95% dimethylpolysiloxane) column. The program began with an oven temperature of 50 °C for 5 min, which was increased by 5 °C/min up to 280 °C, then finally, it was increased by 25 °C/min up to 380 °C. The MS operated with an ionization energy of 70 eV and 250 °C in SCAN mode, with a scanning speed of 1 mL/min, from *m/z* 50 to 500. The detector signal was processed with Environmental ChemStation software (Agilent Technologies, Palo Alto, CA, USA), which allowed automatic identification of the mass spectra generated by automatically comparing them to spectra catalogued in the National Library of Standards and Technology Institute (NIST11), Palo Alto, CA, USA. The purity of each of the signals (peaks) of the chromatograms was checked. Only signals with a purity of one and the identification of spectra with agreement above 90% were accepted.

### 2.3. Antioxidant Capacity of Hexanic Extract Evaluated In Vitro 

#### 2.3.1. Total Antioxidant Activity Determination (Phosphomolybdate Assay)

The total antioxidant activity of the hexanic extract was determined according to the procedure described by Prieto et al. [11]. Briefly, ascorbic acid (0.3 mg/mL; control) or hexanic extract at different concentrations (0.3, 1.0 and 10.0 mg/mL) was added to 100 µL of deionized water and 3 mL of reagent solution (0.6 M sulfuric acid, 28 mM sodium phosphate and 4 mM ammonium molybdate). The reaction mixture was incubated in a boiling water bath for 90 min, and after cooling the absorbance was measured at 695 nm in a Perkin Elmer Lambda 18 UV VIS Spectrophotometer (PerkinElmer Inc., Shelton, CT, USA). The inhibitory activity was calculated with a negative control.

#### 2.3.2. DPPH Assay

Anti-radical scavenger activity was assayed by employing the free-radical compound, 2,2-diphenyl-1-picrylhydrazyl (DPPH•). In brief, the hexanic extract (0.3, 1.0, or 10.0 mg/mL) was added to 1 mL of water, and then 1 mL of DPPH (0.2 mM in ethanol) was added. The solution was mixed and incubated for 30 min in the dark. The absorbance of the mixture was measured at 517 nm in a Shimadzu UV-2550 UV/VIS Spectrophotometer (Shimadzu, Kyoto, Japan). Ascorbic acid (0.3 mg/mL) was used as the positive control [12].

### 2.4. Antioxidant Properties of Hexane Extract Evaluated in Yeast 

*Saccharomyces cerevisiae* strain W303-1A (American Type Culture Collection, Rockville, MD, USA) was cultured in 1% yeast extract, 2% peptone and 2% dextrose (YPD 2%) medium.

#### 2.4.1. Treatment of Yeast Cells

In the mid-long growth phase, 10^6^ cells/mL were inoculated in fresh medium until the stationary phase was achieved. Then, yeast cells were treated with different concentrations of the hexanic extract (0.3, 1.0, and 10.0 mg/mL) or ascorbic acid (0.3 mg/mL, control) and incubated for 30 min at 30 °C with shaking (150 rpm). Next, 2.5 mM H_2_O_2_ was added to the medium to induce oxidative stress, and yeast cells were incubated for 1 h at 30 °C, with shaking at 150 rpm [13].

#### 2.4.2. Viability Determination in Yeast Cells

Cell viability was measured with the 3-(4,5-dimethylthiazol-2-yl)-2,5-diphenyltetrazolium bromide (MTT) assay. After treatment, cells were washed twice with phosphate buffered saline (PBS; pH 7.4), resuspended in fresh YPD (2%), mixed with 150 µL of MTT (5 mg/mL), and incubated under constant stirring for 2 h. Then, each tube was mixed with 1.5 mL of propan-2-ol containing 40 mM HCl. The mixture was vigorously vortexed to release MTT-formazan from the cells, and centrifuged at 3000× *g* for 10 min. The absorbance of the supernatant was measured at 570 nm in a Perkin Elmer Lambda 18 UV VIS Spectrophotometer [14].

#### 2.4.3. Lipid Peroxidation Determination in Yeast Cells

Cells were pelleted at 3000× *g* for 5 min, and the pellet was washed twice with deionized water. The samples were lysed with six cycles of 20-s agitations on a vortex, followed by a 20-s incubation on ice. Lysates were mixed with an equal volume of glass beads in PBS (pH 7.4) and 10% (*w/v*) trichloroacetic acid, followed by centrifugation at 2000× *g* for 3 min. Next, 100 µL of the supernatant was mixed with 0.1 mL of 100 mM EDTA and 0.6 mL of 1% (*w/v*) thiobarbituric acid in 50 mM NaOH. The reaction mixture was incubated in a boiling water bath for 15 min and centrifuged at 3000× *g* for 5 min. The absorbance was measured at 532 nm in a Perkin Elmer Lambda 18 UV VIS Spectrophotometer. Lipid peroxidation was calculated based on the absorbance, with 156 mM^−1^ cm^−1^ as the molar extinction coefficient [15]. The results were normalized according to the supernatant protein content. The protein content was assayed routinely by a modification of the Biuret procedure [16] using bovine serum albumin as the standard.

#### 2.4.4. Protein Carbonylation Determination in Yeast Cells

The content of protein carbonylation was assayed spectrophotometrically, according to Levine et al. [17]. Briefly, 0.3 mg of protein was incubated with 0.2% 2,4-dinitrophenylhydrazine (DNPH) dissolved in 2 M HCl for 1 h. The proteins were precipitated by centrifugation at 5000× *g* and washed with a mixture of ethanol-ethyl acetate (1:1) to remove DNPH. The DNPH-coupled carbonylated proteins were measured at 360 nm in a Perkin Elmer Lambda 18 UV VIS Spectrophotometer. The carbonylated protein content was calculated based on the absorbance, with 22,000 M^−1^ cm^−1^ as the molar extinction coefficient.

### 2.5. Properties of Hexane Extract Evaluated in Experimental Animals

All animal procedures were performed in accordance with the recommendations of the Mexican Federal Regulations for the Use and Care of Animals (NOM-062-ZOO-1999, Ministry of Agriculture, Mexico). All protocols were approved by the Institutional Committee for Use of Animals of the Universidad Michoacana de San Nicolás de Hidalgo (folio 08/2018).

Male Wistar rats (270–330 g) were maintained in a room under conventional environmental conditions, with a typical 12-h light/dark cycle and free access to food and water. Diabetes was induced in rats after a 16-h fasting period with a single intraperitoneal injection of streptozotocin (STZ) (45 mg/kg), freshly dissolved in 0.1 M citrate buffer (4.5 pH). Control rats were injected with the citrate (vehicle) solution. Five days after streptozotocin administration, blood glucose levels were determined using commercial glucometer (Accu-Check Performa System, Roche Diagnostics GmbH, Mannheim, Germany) to confirm hyperglycemia. Rats that exhibited blood glucose levels above 300 mg/dL were included in the diabetic rat study groups. 

Thirty male Wistar rats were divided randomly into 6 groups (*n* = 5) as follows: group 1: control rats (vehicle, 10% ethanol); group 2: diabetic rats (vehicle, 10% ethanol); group 3: NEC3 (normoglycemic rats treated with 3 mg/kg EC); group 4: NEC30 (normoglycemic rats treated with 30 mg/kg EC); group 5: DEC3 (STZ–diabetic rats treated with 3 mg/kg EC); and group 6: DEC30 (STZ–diabetic rats treated with 30 mg/kg EC). All treatments began 2 weeks after injection of STZ or citrate. Rats received treatments daily in the morning by oral gavage for 7 weeks. During that period, their blood glucose concentrations were determined from the tail veins with a commercial glucometer (*Accu-Check Performa*, Roche, Diagnostics GmbH, Mannheim, Germany), and weights were measured weekly.

#### 2.5.1. Tissue Preparation

After 7 weeks of treatment, rats were euthanized. The liver, kidneys, and brain were rapidly excised and placed in ice cold PBS (pH 7.4) and stored immediately at −70°C. For analyses, tissues were weighed, and 25% homogenates were prepared with a *Potter–Elvehjem* homogenizer. Tissue homogenates were used to estimate lipid peroxidation, protein carbonylation, catalase activity and ROS production.

#### 2.5.2. Lipid Peroxidation Determination in Rats

Lipid peroxidation was determined according to a method reported previously [18]. Briefly, 0.5 mg/mL of protein from tissue homogenates was resuspended in 0.1 M phosphate buffer (pH 7.4), mixed with reagent solution (0.375% thiobarbituric acid (TBA), 15% trichloroacetic acid and 0.25 M HCl), and incubated for 15 min in a boiling water bath. After cooling, the flocculent precipitate was centrifuged at 6300× *g* for 5 min. The absorbance was measured at 532 nm in a Perkin Elmer Lambda 18 UV VIS Spectrophotometer 1. Lipid peroxidation was calculated based on the absorbance, with 156 mM^−1^ cm^−1^ as the molar extinction coefficient. 

#### 2.5.3. Protein Carbonylation Determination in Rats

The protein carbonylation content in tissue homogenates was assayed as described above for yeast cells (Section 2.4.4).

#### 2.5.4. Catalase Activity Determination in Rats

Catalase activity was assayed by measuring the conversion of hydrogen peroxide to oxygen with a Clark-type oxygen electrode connected to a biological oxygen monitor (5300A Biological Oxygen Monitor, YSI, Ohio, USA), according to a method reported previously [19]. In brief, 0.5 mg/mL of protein from tissue homogenates was resuspended in 0.1 M phosphate buffer (pH 7.4) at 25 °C and monitored for 1 min. Then, 5 mM H_2_O_2_ was added to the chamber, and the conversion of hydrogen peroxide to oxygen was measured with the oxygen electrode for 3 min. The reaction was stopped with 1 mM sodium azide. 

#### 2.5.5. Determination of ROS Production

Reactive oxygen species production was determined with the fluorescence probe 2’,7’-dichlorodihydrofluorescein diacetate (H_2_DCFDA). Briefly, 0.3 mg/mL of protein from tissue homogenates was resuspended in buffer (10 mM HEPES, 100 mM KCl, 3 mM MgCl_2_, and 3 mM KH_2_PO_4_, pH 7.4) and incubated with 12.5 µM H_2_DCFDA for 15 min in an ice bath with shaking [20]. Basal fluorescence was recorded after 1 min; then, 5 mM glutamate plus malate was added. Changes in fluorescence were recorded for 60 min at excitation/emission wavelengths of 485 nm/520 nm in a Shimadzu RF-5301PC Spectrofluorophotometer (Shimadzu, Kyoto, Japan). 

### 2.6. Statistical Analysis

Results are expressed as the mean ± standard error (SE). Statistical analyses were performed with one-way or two-way analysis of variance (ANOVA) calculations provided with GraphPad Prism (Version 7) software (GraphPad Software, San Diego, CA, USA). *p*-values *p* < 0.05 were considered statistically significant.

## 3. Results

### 3.1. Major Compounds 

**Figure 1:**
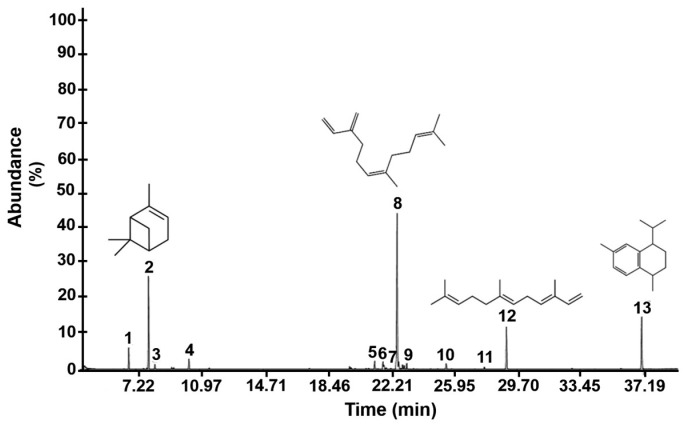
Chromatogram of the hexanic extract of *E. carlinae* inflorescences. (1) α-Pinene; (2) β-Pinene; 3) β-Mircene; 4) γ-Terpinene; 5) α-Bergamotene; 6) (E)β-Farnesene; 7) α-Cariofilene; 8) Zβ-Farnesene; 9) Eudesmadiene; 10) Carotol; 11) α-Bergamotol; 12) α-Farnesene; 13) Calamanene.

The major compounds of the hexanic extract were determined with GC/MS. We identified 13 major compounds (Table 1). The most abundant compound was (Z)β-Farnesene (38.79%), followed by β-pinene (17.53%), calamenene (13.3%), and α-pinene (4.06%). Figure 1 shows the profile of identified compounds.

### 3.2. Antioxidant Activity In Vitro

The total antioxidant activity, assayed with phosphomolybdate, showed that hexanic extract concentrations of 0.3 and 1.0 mg/mL had lower antioxidant activities (14.76 ± 0.42% and 22.92 ± 0.80%, respectively) than the 100% ascorbic acid control. In contrast, the 10.0 mg/mL hexanic extract had higher antioxidant activity (134.1 ± 2.34%) than the control (Table 2). At all concentrations, the hexanic extract displayed lower DPPH• scavenging activity than the control (Table 2). Increasing the extract concentration increased only the total antioxidant activity. Moreover, in a previous work, we found that 30 mg/mL hexanic extract of EC had the same anti-DPPH• activity as the control [10].

### 3.3. Antioxidant Activity in S. cerevisiae (Yeast Cells)

Next, we assayed the antioxidant activity of the hexanic extract of EC in a biological system. We exposed *S. cerevisiae* cells to different concentrations of the hexanic extract, under stress conditions induced by H_2_O_2_ (Table 3). In the presence of H_2_O_2_ alone, *S. cerevisiae* cells showed reduced cell viability (26.06% ± 2.02) compared to 100% viability in control cells. This reduced viability is partially prevented with the addition of ascorbic acid (positive control) or the hexanic extract (0.3, 1.0 and 10.0 mg/mL). However, differences between the effects of ascorbic acid and hexanic extract were not significant at all concentrations. To determine more precisely the capacity of the hexanic extract to protect cells against H_2_O_2_, we measured oxidative modifications in the cells (lipid peroxidation and protein carbonylation) after H_2_O_2_ exposure. Cells exposed to H_2_O_2_ showed a significant increase in lipid peroxidation and protein carbonylation compared to unexposed cells. These increases in oxidative modifications were prevented completely in the presence of ascorbic acid or hexanic extract at all concentrations (Table 3).

### 3.4. Effect of Hexanic Extract on Body Weight and Blood Glucose in Rats

To determine the in vivo efficacy of the hexanic extract of EC, we tested rats with or without diabetes. Diabetes was characterized by an increase in blood glucose and low weight gain. Rats were orally administered two doses of the hexanic extract (3.0 or 30 mg/kg of body weight (BW)) as shown in Table 4. After a 7-week treatment with vehicle, the mean blood glucose in the diabetic group was six times higher (530.3 ± 16.74 mg/dL) than that in the control group (85.6 ± 5.40 mg/dL). However, body weight was lower in the diabetic group than in the control group (400.6 ± 24.22 g and 289.5 ± 11.27 g, respectively). At the end of the 7-week treatment with the lowest dose of hexanic extract (3.0 mg/kg of BW) we observed no impact on blood glucose levels in the NEC3 or DEC3 group. However, at the higher dose (30 mg/kg of BW), we found a significant reduction in blood glucose in the DEC30 group. This change was also associated with a moderate increase in BW. In contrast, neither blood glucose nor BW changed significantly in the NEC3 or NEC30 group (Table 4).

### 3.5. Effect of the Hexanic Extract on Lipid Peroxidation and Protein Carbonylation in Tissue Homogenates 

**Figure 2:**
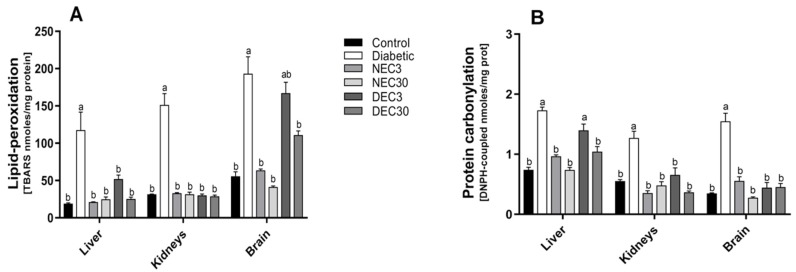
*Eryngium carlinae* extract effects on oxidative modifications in the liver, kidneys, and brain. The results represent the means ± SE of three independent experiments of the (**A**) lipid peroxidation effect and (**B**) protein carbonylation content. Different letters indicate significant differences (*p* ≤ 0.05) between treatments, based on two-way ANOVAs with Tukey’s post hoc test. NEC: normoglycemic + 3 mg of EC; NEC30: normoglycemic + 30 mg of EC; DEC3: diabetic + 3 mg of EC; DEC30: diabetic + 30 mg of EC. TBARS: TBARS: thiobarbituric acid reactive substances; DNPH: dinitrophenylhydrazine.

Tissue lipid peroxidation levels were assayed by measuring thiobarbituric acid reactive substances (TBARS, Figure 2A). The diabetic group exhibited increased lipid peroxidation in liver, kidney and brain compared to the control group. The *E. carlinae* extract did not affect lipid peroxidation in the control group. In contrast, increases in lipid peroxidation due to diabetes were reversed with oral administration of EC extract in all tissues, particularly at the highest dose (30 mg/Kg of BW). Similarly, the highest dose reversed the increases in protein carbonylation caused by diabetes (Figure 2B).

### 3.6. Effect of the Hexanic Extract on Catalase Activity in Tissue Homogenates

**Figure 3:**
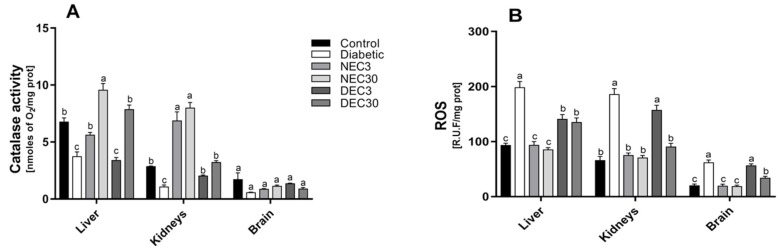
Effects of hexanic extract of *E. carlinae* on oxidative enzyme activity in rat tissues. (**A**) Catalase activity and (**B**) reactive oxygen species (ROS) production were determined in tissue homogenates. The results represent the means ± SE of three independent experiments. Different letters indicate significant differences between treatments *p* ≤ 0.05, two-way ANOVAs with Tukey’s post hoc test. NEC: normoglycemic + 3 mg of EC; NEC30: normoglycemic + 30 mg of EC; DEC3: diabetic + 3 mg of EC; DEC30: diabetic + 30 mg of EC.

Catalase activity was significantly lower in diabetic rats than in control rats in liver and kidney tissues (Figure 3A). The hexanic extract of EC restored catalase activity in diabetic rats.

### 3.7. Effect of the Hexanic Extract on Total Reactive Oxygen Species Production in Tissue Homogenates

In the absence of treatment, the diabetic rat group exhibited higher levels of total ROS than the normoglycemic control group (Figure 3B). Neither dose (3 and 30 mg/kg of BW) of hexanic extract of EC altered the total ROS in normoglycemic rats. Similarly, diabetic rats treated with the lowest dose showed no significant reductions in ROS in kidney or brain tissues. However, the administration of 30 mg/kg of BW of the hexanic extract of *E. carlinae* in diabetic rats reduced ROS levels below the levels observed in the untreated diabetic group in all tissues. Nevertheless, ROS levels remained higher than those observed in the normoglycemic control group, in the brain and liver, but not in the kidneys.

## 4. Discussion 

Studies have shown that distinct species of the genus *Eryngium* demonstrated antioxidant activities [6,21]. In the present study, we found that the main compounds extracted from *E. carlinae*, with *n*-hexane as a solvent, were terpenes and sesquiterpenes, which were previously shown to possess antioxidant properties [22]. Farnesene, the major compound in the extract (Table 1), is an acyclic hydrocarbon sesquiterpene (C_15_H_24_). Farnesene is primarily insecticidal; it is innocuous in plants, higher-order animals, and man. The inflammatory and anti-carcinogenic activities of various plants, such as anise, chamomile, hops, lavender, coriander, ginger, apples, and carrot seeds, were attributed to the presence of farnesene. More recently, farnesene was shown to have in vitro antioxidant effects [22,23]. β-Pinene (C_10_H_16_), another one of the major components of the hexane extract, is a monoterpene bicyclic compound present in the vast majority of essential oils that possesses antioxidant activity [21,24]. Moreover, calamenene (C_15_H_22_) and its analogs were previously correlated with antioxidant activity in vitro and antimicrobial activity in plants, such as damiana, guava, and sage [25,26]. Indeed, the antioxidant activity of a compound, either pure or within a complex mixture, is based on the ability to donate hydrogen atoms or electrons and to chelate metal cations or radicals, which reduces their potentially harmful effects [27]. In addition, monoterpenes hydrocarbons, which are present in the hexane extract of *E. carlinae*, are those with strongly activated methylene groups in their structure that were the most active. In relation to sesquiterpenes groups, also present in the hexane extract of *E. carlinae*, the radical scavenging properties of the hydrocarbons-type were quite low and lower than that of the monoterpene hydrocarbons group, whereas among the oxygenated type, mainly allylic alcohols showed good scavenging properties, similar to those of oxygenated monoterpenes [28]. In several plants, this in vitro antioxidant activity was shown to result from the presence of terpenes and sesquiterpenes, consistent with our findings in the *E. carlinae* extract (Table 1). 

*Saccharomyces cerevisiae* is a simple eukaryotic organism with systems that are significantly homologous to mammalian systems, including conserved metabolic pathways. The simplicity in obtaining and handling yeast makes it a convenient model for analyzing the mechanisms and responses to oxidative stress, by measuring various biomarkers of oxidative stress [29,30,31]. This experimental model is appropriate for assaying natural products or medicinal plant extracts [32]. Previous studies have shown that yeast was useful for investigating aging and metabolic stress limited to several cellular organelles in the cytosol, vacuoles, or mitochondria [33,34,35]. In the present study, we used yeast in the stationary state, which was correlated to the quiescent state, which occurs in the natural life of the majority of both unicellular and multicellular eukaryotic cells [31,36]. However, when yeast is affected by adverse conditions, functional damage occurs to biomolecules, such as membrane lipids, proteins, and nucleic acids. This damage occurs through the production of ROS, which affects lipid peroxidation, protein carbonylation, and/or nucleic acid oxidation [13,37,38]. In this study, both lipid peroxidation, and protein carbonylation induced by ROS were diminished by the antioxidant activity of *E. carlinae* extract.

## 5. Conclusions

The kidneys and brain are the major organs targeted in the diabetic milieu mainly by glycation, a nonenzymatic reaction between reducing sugars and proteins [39]. Currently, we found that the hexanic extract of *E. carlinae* inflorescences has significant hypoglycemic and hypolipidemic properties in hearts of diabetic rats [10]. An increase in the levels of glucose in a sustained manner is a key factor in brain damage. In addition, oxidative stress and the generation of ROS are associated with brain damage during diabetes; likewise, oxidative damage has been correlated with the deterioration of cognitive functions [40,41]. Furthermore, hyperglycemia caused structural damage, oxidants and functional damages to kidneys [42]. The liver is another organ that highlights the most recent basic and clinical data underlying the development of diabetes where fatty liver and diabetes share insulin resistance as their pathogenic determinant [43]. In this study the hexanic extract of inflorescences of *E. carlinae* have a protective effect by reducing the oxidizing damage both in the brain, kidneys and liver of diabetic rats. In conclusion, this study demonstrated that the hexanic extract of *Eryngium carlinae* mainly comprised (Z)β-Farnesene, β-pinene, and calamenene, and that this extract exerted antioxidant activity. These antioxidant activities diminished the peroxidation of lipids in vitro, increased viability in *S. cerevisiae*, and ameliorated hyperglycemia and oxidative damage in diabetic rats.

## Figures and Tables

**Table 1:** Major compounds of the hexanic extract of *Eryngium carlinae* inflorescences.

Compound	Retention Time (min)	Abundance (%)	Compound	Retention Time (min)	Abundance (%)
α-Pinene	6.79	4.06	(Z)-beta-Farnesene	22.64	38.79
β-Pinene	7.96	17.53	Eudesmadiene	22.73	1.73
β-Mircene	8.34	1.0	Carotol	25.53	1.40
γ-Terpinene	10.34	2.23	α-Bergamotol	27.79	0.48
α-Bergamotene	21.31	1.92	α-Farnesene	29.09	10.38
(E)-β-Farnesene	21.8	1.65	Calamenene	37.08	13.30
α-Caryophyllene	21.86	0.95			

**Table 2:** Antioxidant activity of the hexanic extract of *E. carlinae* inflorescences in vitro.

Concentration (mg/mL^−1^)	% TAA (Mean ± SE)	% Anti-DPPH• Activity
Ascorbic acid 0.3	100	92.87 ± 0.09
Hexanic extract 0.3	14.76 ± 0.42 ***	52.24 ± 0.29
1.0	22.92 ± 0.80 ***	52.41 ± 0.30 ***
10.0	134.1 ± 2.34 ***	55.64 ± 0.34 ***

Results are expressed as the mean ± SE of three independent experiments. *** *p* < 0.001, one-way ANOVA with Dunnett’s post hoc test. TAA: total antioxidant activity; anti-DPPH radical activity: radical scavenging activity; SE: standard error.

**Table 3:** Effects of the hexanic extract of *E. carlinae* on W303-1A yeast viability and oxidative modifications.

Condition	% Viability (% control) (Mean ± SE)	Lipid-Peroxidation TBARS [nmoles/mg prot] (Mean ± SE)	Protein Carbonylation [nmoles/mg prot] (Mean ± SE)
Control	100	15.33 ± 0.423	1.028 ± 0.135
2.5 mM H_2_O_2_	26.06 ± 2.021	32.98 ± 0.526 ***	1.975 ± 0.116 **
Ascorbic acid 0.3 + 2.5 mM H_2_O_2_	50.54 ± 1.453 ***	10.87 ± 0.370 ***	0.918 ± 0.065 *
Hexanic extract of EC 0.3 + 2.5 mM H_2_O_2_	44.37 ± 1.824 **	15.81 ± 0.428	1.346 ± 0.083
1.0 + 2.5 mM H_2_O_2_	51.67 ± 1.818 ***	11.15 ± 0.418 **	1.003 ± 0.079
10.0 + 2.5 mM H_2_O_2_	69.85 ± 3.248 ***	11.01 ± 0.435 **	0.926 ± 0.033 *

Results are expressed as the mean ± SE of three independent experiments. *: *p* < 0.05, **: *p* < 0.01, ***: *p* < 0.001, according to one-way ANOVA with Dunnett’s post hoc test. TBARS: thiobarbituric acid reactive substances; EC: *E. carlinae* extract; nmoles/mg prot: nanomoles/mg protein.

**Table 4:** Effect of the hexanic extract of *E. carlinae* inflorescences on body weight and blood glucose.

Group	Blood Glucose (mg/dL)		Body Weight (g)	
	Initial (Mean ± SE)	Final (Mean ± SE)	Initial (Mean ± SE)	Final (Mean ± SE)
Control	91.6 ± 3.89 ^b^	85.6 ± 5.40 ^c^	298.9 ± 11.27	400.6 ± 24.22 ^a^
Diabetic	521.2 24.93 ^a^	530.3 ± 16.74 ^a^	308.8 ± 3.381	289.5 ± 7.469 ^b^
NEC3	85.8 ± 5.62 ^b^	86.6 ± 3.25 ^c^	296 ± 6.138	409.4 ± 12.25 ^a^
NEC30	83.2 ± 4.33 ^b^	90.2 ± 5.27 ^c^	290.2 ± 8.11	382 ± 15.66 ^a^
DEC3	514.4 ± 9.07 ^a^	558.1 ± 15.8 ^a^	313.7 ± 3.40	290.4 ± 10.02 ^b^
DEC30	518.8 ± 24.01 ^a^	410 ± 40.38 ^b^	315.8 ± 3.53	304.2 ± 19.71 ^b^

Different letters indicate significant differences (*p* ≤ 0.05) between treatments, based on two-way ANOVAs with Tukey’s post hoc test. NEC: normoglycemic + 3 mg of EC, NEC30: normoglycemic + 30 mg of EC, DEC3: diabetic + 3 mg of EC, DEC30: diabetic + 30 mg of EC.
